# Angioimmunoblastic T-Cell Lymphoma: A Questionable Association with Follicular Dendritic Cell Sarcoma

**DOI:** 10.1155/2017/9601094

**Published:** 2017-01-18

**Authors:** Daniel Benharroch, Miriam Zekzer, Karen Nalbandyan

**Affiliations:** ^1^Department of Pathology, Soroka University Medical Center and Faculty of Health Sciences, Ben-Gurion University of the Negev, Beer-Sheva, Israel; ^2^Department of Hematology, Soroka University Medical Center and Faculty of Health Sciences, Ben-Gurion University of the Negev, Beer-Sheva, Israel

## Abstract

An elderly woman presented with generalized lymphadenopathy, several systemic symptoms, and splenomegaly. An inguinal lymph node excision revealed a compound picture. One aspect of the lymph node morphology, including cells with follicular T-helper cell phenotype, was most consistent with angioimmunoblastic T-cell lymphoma. The other component, revealing spindle cells forming whorls with immunostaining for CD21, CD23, and fascin, might be an integral part of this T-cell lymphoma. However, due to the often massive involvement of the nodal tissue by these follicular dendritic cells, these areas were questionably suggestive of involvement by follicular dendritic cell sarcoma. We raise herein the issue of the borderline area between advanced follicular dendritic cell expansion in angioimmunoblastic T-cell lymphoma and a massive follicular dendritic cell proliferation consistent with follicular dendritic cells sarcoma, in the absence of a genomic analysis.

## 1. Introduction

Angioimmunoblastic T-cell lymphoma (AITL), a rare aggressive malignant tumor located predominantly in lymph nodes, presents usually with several systemic symptoms. On histology, this neoplasm shows a polymorphic infiltrates of T-cells, with variable components of B-immunoblasts, eosinophils, and plasma cells. Moreover, the tumor T-cells demonstrate the immunophenotypic features of follicular T-helper cells. In addition, the proliferation of high endothelial venules, some arborizing, is prominent. In some cases, poorly delimited follicular hyperplasia is found. In most instances, a variable follicular dendritic cell (FDC) meshwork expansion is evident. Although the expanding FDCs have been extensive at times, the documentation of these FDC proliferation instances is strikingly modest. We raise the query of an expansion which is excessive to a degree that it elicits a picture consistent with a genuine FDC neoplasm.

Follicular dendritic cell sarcoma (FDCS) is a very rare entity affecting usually lymph nodes, mainly cervical and abdominal. This disease belongs to the histiocytic and dendritic cell category of malignancies. It presents mostly as an indolent tumor and is rarely more aggressive [1, 2].

Composite FDCS have been described in conjunction with follicular lymphomas [3] and chronic lymphocytic leukemias/small lymphocytic lymphomas (CLL/SLL) [4] and with Castleman disease [5].

We presently report on a T-cell lymphoma, most consistent with angioimmunoblastic T-cell lymphoma (AITL) [6, 7], occurring in an 80-year-old woman's inguinal lymph node. Since the FDC component was often massive, we suggest the possible differential diagnosis of a composite AITL with follicular dendritic cell sarcoma. The patient died of the malignant tumor 5 months after diagnosis, perhaps independent of her objecting to chemotherapy.

## 2. Report of a Case

This 80-year-old Jewish woman from India was referred to the hematologist for high fever, night sweats, weight loss, itching, multiple lymphadenopathy, and splenomegaly. She had been previously diagnosed as suffering from polyarthropathy, suggestive of rheumatoid arthritis. She was also hospitalized three weeks before diagnosis with a bout of necrotizing enterocolitis and reacted to the Flagyl® therapy for* Clostridium difficile* with a rash. She had been also followed for alcoholism for several years.

The patient was found to be anemic and thrombocytopenic; LDH was 770. A CT scan showed enlarged lymph nodes at most sites, the largest in the axillae. Excision of a superficial lymph node, from the left inguinal area, was performed.

**Figure 1:**
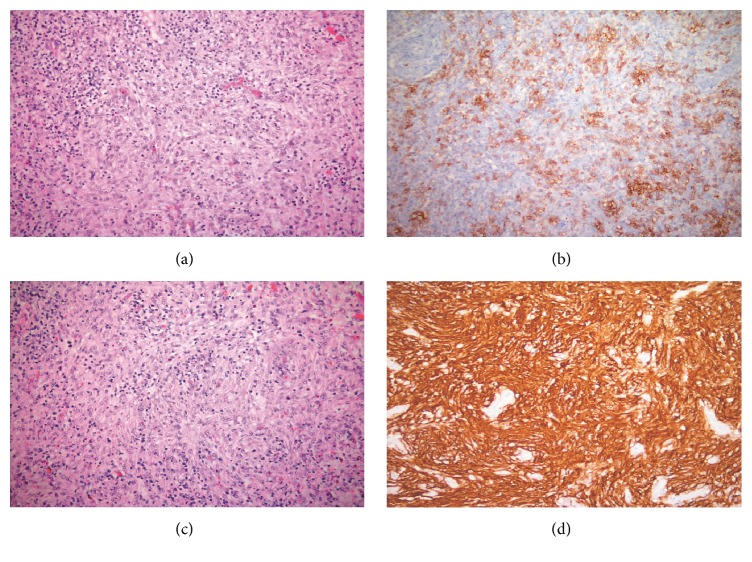
(a) Both components of the histologic picture are seen in this section. Small-to-medium-sized lymphoid cells with clear cytoplasm and eosinophils are segregated by intervening whorls of spindly follicular dendritic cells (H&E ×320). (b) Numerous PD1+ cells are found in the cellular area of the lesion consistent with AITL (Immunohistochemistry with DAB ×320). (c) Several whorls of follicular dendritic cells are prominent in this section (H&E ×320). (d) The spindle follicular dendritic cells show very strong CD21 immunostaining. Note the negative staining related to the high endothelial venules (Immunohistochemistry with DAB ×320).

On low power microscopy, the lymph node structure was effaced and showed two distinct types of histological features (Figure 1(a)). The first consisted of highly cellular areas, composed of small-to-medium-sized cells, often with a clear cytoplasm; these were predominantly T-cells: CD3+++; CD2+++; CD4+++; CD8++; CD5+++; CD7+/(−); PD1++ (Figure 1(b)), in a polymorphous cellular background, including eosinophils and isolated large CD20+ cells. In addition, the tumor cell phenotype included CD10+++; CD68+; LCA+; CD30 (−); Ki67+ 75%. Relatively few high endothelial venules were noted. A PCR for TCR revealed two highly reproducible clonal peaks, confirming the evidence of a T-cell lymphoma.

The T-cell lymphoma component which showed CD4+++; CD10+++; PD1++; Bcl-6++, suggestive of the follicular T-helper cell phenotype, was most consistent with AITL.

The other aspect of the histology was composed of spindle cells with small uniform nuclei, arranged in whorls formations (Figure 1(c)). These cells were CD21+++ (Figure 1(d)); CD23+++; fascin ++; HLA-DR++. This FDC component was excessive, even relating to the description of the “extensive FDC network expansion”, most certainly when comparing our patient lymph node with those documented in the literature. Though no genomic analysis was available, the morphologic features, together with the immunophenotype, may be suggestive of a low-grade follicular dendritic cell sarcoma.

A bone marrow biopsy was infiltrated by small and medium cells positive for T-cell markers. No FDC were identified. Involvement of the bone marrow by the T-cell lymphoma was suggested.

The patient was treated with corticosteroids for several weeks, and, at first, the symptoms and the lymphadenopathy receded. However, the improvement soon gave way to a flare-up. At all times, the patient totally declined the use of chemotherapy.

The patient condition deteriorated rapidly. She died 5 months after the diagnosis was established. The request for an autopsy was rejected by the family.

## 3. Discussion

Follicular dendritic cell sarcoma (FDCS) is a very rare tumor developing mainly in lymph nodes. It is one of the histiocytic and dendritic cell neoplasms, which have a common phagocytic or antigen-processing capacity but do not represent a single cell of origin. Data on this tumor are scarce. Only infrequently is the tumor aggressive. Rarely, it has occurred in conjunction with another lesion, like Castleman disease, mainly of the hyaline-vascular type. Moreover FDCS has occurred with malignant tumors, notably with follicular lymphoma or CLL/SLL.

The pathogenesis of FDCS is unknown. In the composite form with Castleman disease, the neoplasm probably arises from regressive germinal centers, which contains little else than atypical follicular dendritic cells. In follicular lymphoma, the tumor follicles may include also various amounts of follicular dendritic cells. However the pathogenesis here is still questioned.

In AITL, a CD21-positive follicular dendritic expanded meshwork is loosely aggregated mainly around blood vessels. This malignant tumor produces several cytokines and chemokines [8–10]. Thus the T-cells and the blood vessels, mainly postcapillary venules, may be induced to proliferate. In addition, the variably sparse B-immunoblasts in AITL may develop into a large B-cell lymphoma. Hypothetically, follicular dendritic cells (FDCs) may proliferate under the same cytokines/chemokines stimulation [11]. Following the proliferation of the FDC, atypical changes of these cells with increased mitoses may develop as evidence of a neoplasm.

The literature about AITL and the related FDC meshwork proliferation is somewhat intricate. It is stated that this network expansion may be very extensive; however the microphotographic records thereof show much more discreet FDC proliferation instances. Thus one is left with a query relating to the borderline between a benign proliferation and a malignant tumor, at least in terms of the proportion of FDCs in the lymph node, considering the generally low-grade nature of this tumor.

The lymph node of our patient might be involved, then, by a composite AITL associated with FDCS.

Starkey et al. described an elderly male patient with peripheral T-cell lymphoma (PTCL) with an excessive dendritic cell network mimicking follicular dendritic cell tumor [12]. By now, it is clear that PTCL-NOS and AITL show several overlapping features. Although this patient presented without hepatosplenomegaly, or hypergammaglobulinemia, his health was deteriorating because of COPD, and he showed a widespread pruritic rash. Since an expanding FDC network and a T-follicular helper phenotype were described; the overall picture may be more in favor of AITL.

In addition, this case [12] emphasizes the same irresolute attitude between a markedly expanded FDC meshwork and FDCS, as we have suggested in the present case report. In fact, the nodular FDC proliferation [12] is more in favor of FDCS, when compared with our own findings. These authors' reservations were that the FDC network in their patient was entangled with tumor T-cells, which is not the case with our patient. However, aberrant T-cell markers expression in FDCS has later been described [13].

We therefore suggest that the 2006 report [12] is indeed of a composite AITL with FDCS. In contrast, our patient remains in the borderline area of composite AITL with FDCS, while we cannot exclude an AITL with an excessively expanded FDC meshwork, according to existing criteria.

The relationship between the composite tumor and polyarthropathy or rheumatoid arthritis is not clear. On the one hand, lymphoproliferative disorders (LPD) have been described in patients with autoimmune disorders, like rheumatoid arthritis, psoriasis, or dermatomyositis, when treated with methotrexate. These LPD were found to resemble AITL [14]. On the other hand, AITL, among the many symptoms it induces, may rarely present with polyarthropathy suggesting rheumatoid arthritis [15]. Our patient had never been treated with methotrexate. Moreover she had suffered from polyarthropathy several years before she developed the lymphoma.

In addition to the irresolute position on the malignancy of the FDC histological component, we include a further limitation. Some hematologists may have readily established the diagnosis of AITL on the ground of the clinical features only. The lymphoma cell population was clearly composed of follicular helper T-cells and of an important FDC component, but of few high endothelial venules and EBV+, CD20+ immunoblasts. We strongly suggest that the morphologic and phenotypic picture are consistent with the “tumor cell-rich” form of AITL. Nevertheless, the discrimination between AITL and acquainted THF-related PTCL may be an exacting intellectual exercise at this stage of our knowledge [16].

In conclusion, we have described the gloomy progression of a malignant tumor affecting an 80-year-old Indian woman. The complex neoplasm included, on the one hand, AITL, an aggressive and highly symptomatic cancer. On the other hand, the AITL was intermingled with an excess of FDC perhaps amounting to FDCS. A similar occurrence has not been reported to date.
